# Metabolomics-Based Study of Clinical and Animal Plasma Samples in Coronary Heart Disease with Blood Stasis Syndrome

**DOI:** 10.1155/2012/638723

**Published:** 2012-05-20

**Authors:** Huihui Zhao, Jianxin Chen, Qi Shi, Xueling Ma, Yi Yang, Liangtao Luo, Shuzhen Guo, Yong Wang, Jing Han, Wei Wang

**Affiliations:** Department of Graduate School, Beijing University of Chinese Medicine, Beijing 100029, China

## Abstract

The aim of this study is to explore a bridge connecting the mechanism basis and macro syndromes of coronary heart disease with experimental animal models. GC-MS technique was used to detect the metabolites of plasma samples in mini swine models with myocardial infarction (MI) and patients with unstable angina (UA). 30 metabolites were detected in the plasma samples of more than 50 percent of model group and control group in swine, while 37 metabolites were found in the plasma samples of UA patients and healthy control group. 21 metabolites in the plasma samples of swine model and 20 metabolites in patients with UA were found of significant value. Among which, 8 shared metabolites were found of low level expression in both swine model and UA patients. Independent Student's *t*-test, principal component analysis (PCA), and hierarchicalcluster analysis (HCA) were orderly applied to comprehend inner rules of variables in the data. The 8 shared metabolites could take place of the 21 or 20 metabolites in classification of swine model with MI and UA patients, which could be considered as a bridge connecting the mechanism basis and macrosyndromes of swine model with MI and UA patients.

## 1. Introduction

Coronary heart disease (CHD) causes more than one million Chinese to death each year [1]. Unstable angina (UA) is one of the most dangerous types of CHD that has a high mortality and morbidity in the world. Comparing the metabolites in swine model and patients with UA of blood stasis syndrome at the level of metabolomics to explore the underlying mechanism is a new way deserved trying.

Metabonomics, the study of metabolites and their roles in various disease states, is a novel methodology arising from the postgenomics era. In the last decade, “metabonomics” has demonstrated enormous potential in further understanding of many diseases, including work in cardiovascular research [2]. Metabonomics is now recognized as an independently and widely used technique for deriving new biochemical-based assays for disease diagnosis, understanding the relationships between gene function and metabolic control in health and disease, and identifying combination biomarkers for disease [3].

GC (gas chromatography) plays an important role in metabonomics research nowadays. Chromatography has been used mainly in biofluid analysis, especially for target component analysis but not for whole sample profiling combined with chemometrics [4]. The high separation power and the ability to achieve high sensitivity are strong incentives for the consideration of its use in biofluid fingerprinting as well. So chromatography would provide additional and complementary information that cannot be achieved with NMR [5].

In this study, blood samples of UA patients were collected under clinical epidemiology and chemically detected by metabolomics methods. Meanwhile, blood samples of swine model with MI were detected by metabolomics methods. As we had found in former research, feature selection-based data mining methods is more suitable for identifying biomarkers for UA [6]. Alternatively, we combined independent *t*-test and classification-based data mining methods. The novel method presented here provides a better insight into the pathology of a disease.

## 2. Materials and Methods

### 2.1. Animal Sample Preparation

Seventeen male miniswine weighed 25 ± 5 kg were randomly divided into two groups. The model group was composed of nine swine, while the control group was eight. The swine in model group (*n* = 9) was placed with Ameroid constrictor (Research Instrument SW, USA) at the left anterior descending coronary artery to gradually induce chronic myocardial infarction (MI). The swine in control group (*n* = 8) was chest-opened and coronary artery-isolated without placing any constrictor. Based on early results of dynamic observations, evaluations were performed four weeks after operation. Meanwhile, blood was obtained from former cava vena.

### 2.2. Evaluation of Swine Model with MI and Blood Stasis Syndrome

Evaluation of swine with MI and blood stasis syndrome was performed from five parts. Basic phenotype changes, ECG, echocardiography, coronary angiography, and hemorrheology were used to assess the animal model.

**ECG**

More than 0.1 mV ST segment depression and inversion T waves were observed in several leads of surface electrocardiogram of model animals. But no significant arrhythmia was found in experimental period.

**Echocardiography**

The structure and function of left ventricular were evaluated by echocardiography. Compared with sham-operated animals, there was great increase of end-systolic volume (*P* < 0.05), end-diastolic volume (*P* < 0.05), end-systolic diameter (*P* < 0.01), and end-diastolic diameter (*P* < 0.01) in model animals. Anterior wall thickness of both papillary muscle and apex level was decreased at end-diastolic and end-systolic in model animals (*P* < 0.05, *P* < 0.01). Meanwhile, the apex of the left ventricular anterior systolic wall thickening decreased (*P* < 0.01). In addition, septal thickness at the end-systolic decreased (*P* < 0.05). All the results above showed a segmental dysfunction of the left ventricular. There were no significant changes in stroke volume, ejection faction, fraction of shortening, and E/A, which suggested that cardiac function was still in compensatory period.

**Coronary Angiography**

Ex vivo angiography performed more than 90% occlusion or even completely block of the left anterior descending artery by the Ameroid constrictor in model animals. TIMI flow grade I or II was observed in most model animals. There was good filling in the left anterior descending artery with TIMI flow grade III in sham-operated animals.

**Phenotype Information**

Model animals appeared mental stress, irritation, fear, violent behaviors, strong self-defense, pilose disorderly, and lack of luster, while sham-operated animals' performances gradually returned to normal, with increased appetite, neat, and shiny pilose.

**Hemorrheology**

Compared with sham-operated animals, the blood viscosity of model animals increased significantly at a shear rate of high (*P* < 0.05), mid (*P* < 0.01), low (*P* < 0.01).

It was found that swine in MI group had significant changes when compared with the sham operation group from each aspect. Combined the five aspects above, the swine could be diagnosed as MI with blood stasis syndrome.

The results of echocardiography and hemorrheology of mini swine were given in Supplemental Table  1. (see Supplementary material available online at doi:10.1155/2012/638723) 

### 2.3. Clinical Blood Sample Collection

Patients who suffered from UA and blood stasis at Dongzhimen Hospital affiliated to Beijing University of Chinese Medicine were included in the screening of metabolite biomarker cohort study. All patients aged from 55 to 75 were eligible for enrollment. Diagnosis standard of CHD refers to “Treatment Guide of Stable Angina” (ACC/AHA/ACP-ASIM, 1999) and “Diagnosis and Treatment Recommendations of Unstable Angina” (Chinese Society of Cardiology, 2000) [7, 8]. Diagnosis standard of blood stasis syndrome refers to “Guiding principles for the clinical study of Chinese Medicines” (2002) and Standard of syndrome differentiation of coronary heart disease (1990) [9, 10].

Patients were excluded from the case population in four conditions: those who suffered from acute myocardial infarction (AMI), infective cardiomyopathy, cardiac neurosis, or intercostal neuralgia; those who suffered from angina that was caused by polyarthritis rheumatica acuta, great pox, inborn coronary abnormity, hypertrophic cardiomyopathy or aortic valve stenosis; besides UA, those who also suffered from stroke, diabetes mellitus, pulmonary infection, nephritis, renal failure, urinary system infection, rheumatism, or osteoarthrosis; women in pregnant or in lactation.

The control group included healthy people that of no significantly different baseline compared with the case group. After these analyses, blood samples from a total of 27 UA patients with blood stasis syndrome and 15 healthy controls were enrolled for the further metabolomic analysis. The local ethics committee of Beijing University of Chinese Medicine approved the study protocol, and all patients provided written and informed consent.

The demographic details of included subjects were given in Supplemental Table  2.

### 2.4. Gas Chromatography: Mass Spectrometry (GC-MS) Analysis of Human and Animal Plasma

Add 250 *μ*L acetonitrile and centrifuged to 100 *μ*L plasma samples after ice-bathing for 10 minutes. Place 250 *μ*L supernatant plasma extraction liquid in derivation reaction bottle and blew by N2. Add 50 *μ*L methoxylamine pyridine solution with a concentration of 15 mg/mL to uniformly mixed, which was in oximation for one hour and then added by 50 *μ*L derivation reagent (volume ratio of MSTFA : TMCS is 100 : 1) to uniformly mixed for one hour. Add 100 *μ*L skellysolve C containing 0.1 mg/mL docosane to uniformly mixed and then centrifuged for 10 minutes. The resulting clear supernatant liquid was extracted to small volume sample injection vessel for GC-MS analysis, whose conditions were given as following. Temperature of sample injection was 270°C, none-split stream sampling was used, and quantity of injection was 1 *μ*L. Solvent was delayed for 7 minutes. Temperature was initialed at 80°C for 5 minutes and then gradually raised to 300°C with a speed of 10°C/min. It remained at 300°C for 5 minutes. Interface temperature was 280°C, and ion source temperature was 230°C. Ionizing voltage was 70 eV; quadrupole temperature was 150°C. Helium was used as carrier gas with a flow rate of mL/min. 45~550 m/z frequency was used to completely scan the samples.

### 2.5. Data Mining Methods

Independent Student's *t*-test, Principal component analysis (PCA), and hierarchical cluster analysis (HCA) were orderly applied to comprehend inner rules of variables in the data. These methods linearly investigated the data from one variable (*t*-test associated method) to multiple variables (PCA and so on). Moreover, the methods involved unsupervised methods (PCA and HCA). The systematical application of the statistical methods guaranteed the useful information of the disease mined by them.

## 3. Results

### 3.1. Detection of Significantly Different Metabolites by Student's *t*-Test Statistics

Student's *t*-test was initially employed to detect metabolites that are of significant difference between model and sham operation in swine as well as between UA patients and healthy people. 21 metabolites in the plasma samples of swine model, and 20 metabolites in patients with UA were found of significantly value. Among which, 8 shared metabolites between swine model and UA patients were lined in Table 1. The different metabolites were lined in Table 2. Supplemental Table  3 listed the identified compounds and their retention time.

### 3.2. The Results of PCA Analysis

**Figure 1:**
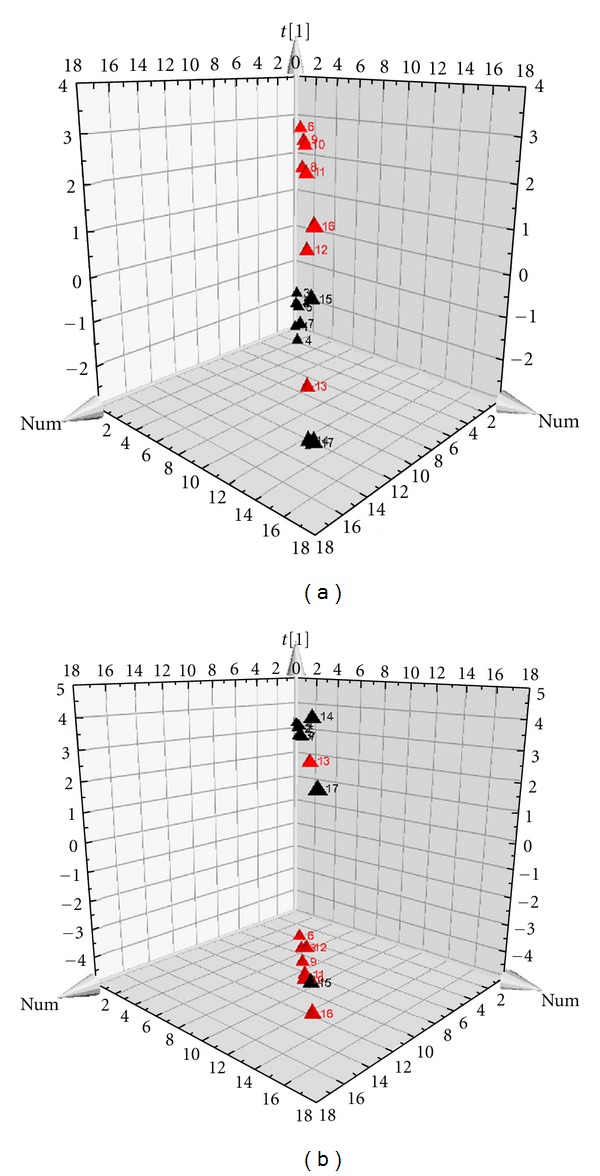
PCA scores' plots of miniswine. (a) is classified by the 21 metabolites in the plasma samples of swine model, and (b) is classified by the 8 same metabolites.

**Figure 2:**
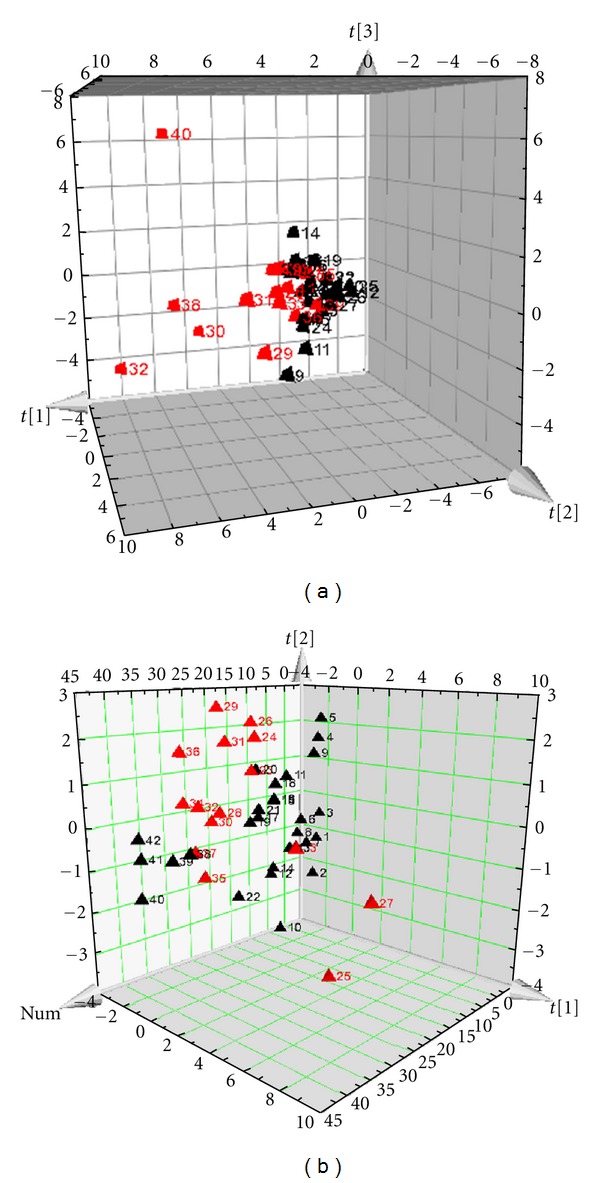
PCA scores plots of UA patients. (a) is classified by the 20 metabolites in the plasma samples of patients with UA, and (b) is classified by the 8 same metabolites. Notes: *X*-axis represents the first component of the metabolite data. *Y*-axis represents the second component, and *Z*-axis represents the third component.

PCA results showed that 21 metabolites classified miniswine model from sham operation group, 20 metabolites classified UA patients from healthy people, moreover, the 8 shared metabolites between swine model and UA patients can distinguish model group from sham operation group in swine and UA patients from healthy people as well. PCA results of miniswine with MI and blood stasis syndrome were showed in Figure 1. PCA results of UA patients with blood stasis syndrome and healthy people were showed in Figure 2.

### 3.3. The Results of Hierarchical Cluster Analysis (HCA)

**Figure 3:**
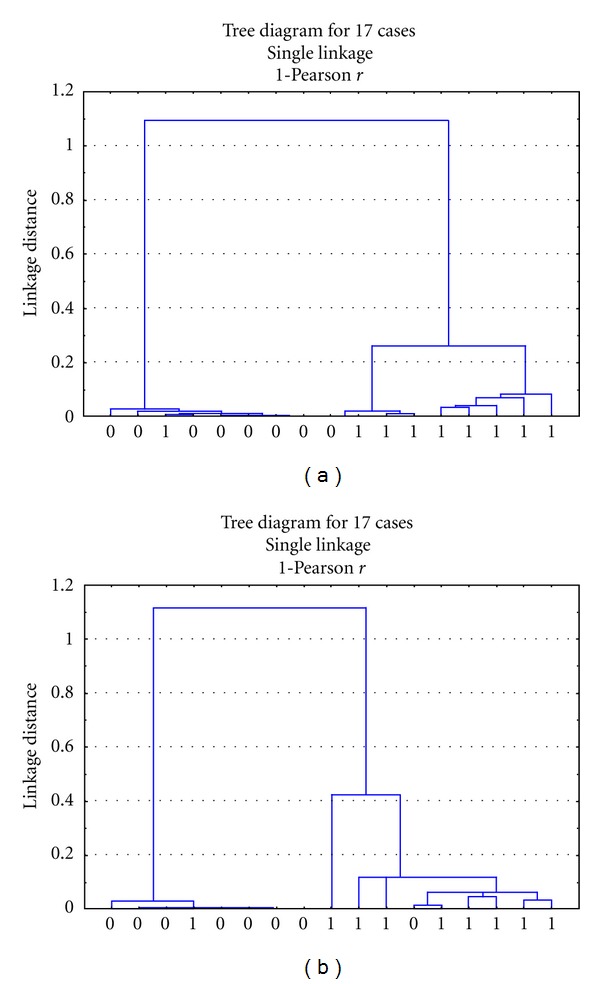
Hierarchical cluster diagrams of miniswine. (a) is classified by the 21 metabolites in the plasma samples of swine model and control, and (b) is classed by the 8 same metabolites. Notes: *X*-axis represents the sample numbers. The *Y*-axis represents the linkage distance (0 = control group;  1 = model group).

**Figure 4:**
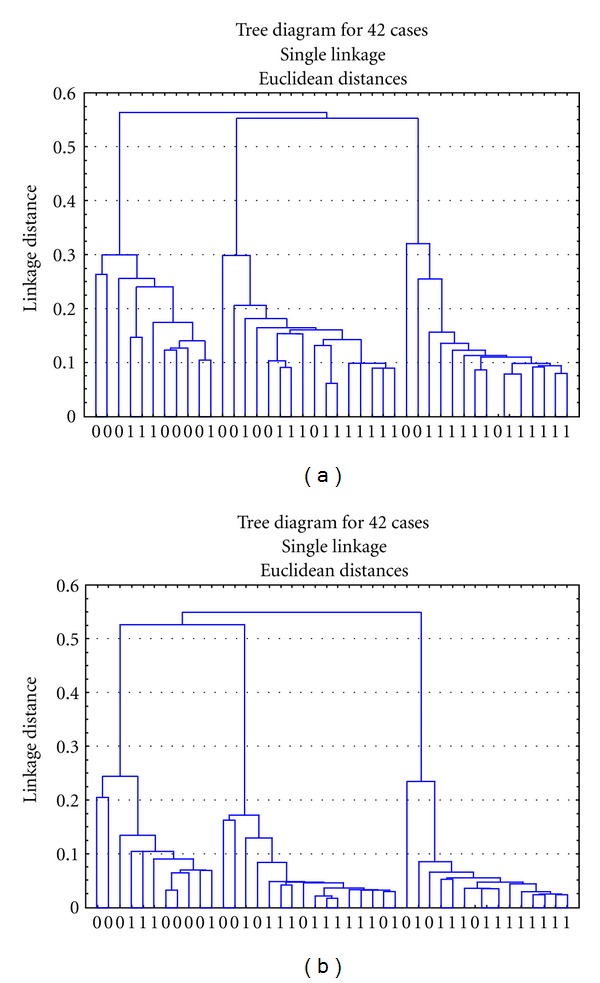
Hierarchical cluster diagrams of UA patients and healthy controls. (a) is classified by the 20 metabolites in the plasma samples of UA patients and healthy controls, and (b) is classified by the 8 same metabolites. Notes: *X*-axis represents the sample numbers. *Y*-axis represents the linkage distance (0 = healthy control group;  1 = UA patients group).

It showed that the 8 shared metabolites are of the same value with the 21 or 20 metabolites in classification of model group from sham operation group in swine and UA patients from healthy people (Figures 3 and 4).

## 4. Discussion

Low level expressions of the eight molecules, 1,4-benzenedicarboxylic acid, 1,5-anhydroglucitol, 2-keto-d-gluconic acid, azelaic acid, heptanedioic acid, pentanedioic acid, ribitol, and serine were detected in swine models as well as in UA patients, which could be considered as a bridge connecting the mechanism basis and macrosyndromes of swine model with MI and UA patients. But further researches need to be carried out, and more evidence needs to be investigated to confirm this hypothesis. Plasma concentration of 1,5-anhydroglucitol decreased in diabetic patients has been confirmed [11, 12]. In order to eliminate interferences, diabetes patients were excluded in our research. Our study found that low expression of 1,5-anhydroglucitol existed in UA patients with blood stasis syndrome as well as in MI swine with blood stasis syndrome. Besides 1,5-anhydroglucitol, the other seven low expressed molecules are newly found. We also found that sugar and fatty acids decreased in UA patients [13]. It indicated that UA may correlate with energy metabolic obstacle and inflammatory reaction. Low level expression of the eight molecules observed in our study may provide an important target for future study. 

GC-MS is mainly used in plant metabonomics [14]. In order to apply GC-MS analysis, samples need to undergo the derivatization process to increase its stability and volatility. Therefore, GC-MS analysis is limited to detect volatile substances and cannot be used to analyze thermal instable substances, such as lipid membrane, or metabolites of high molecular weight. In this study, we used GC-MS to analyze metabolites difference of plasma in swine model and in UA patients. In future study, other metabonomics technology can be used in corporately to examine more metabolites and further explore the underlying mechanisms of UA. Another interesting research is to investigate whether similar metabolic changes also exist in other animal models of coronary heart disease.

## 5. Conclusion

In this study, the plasma samples of UA patients and MI swine model with blood stasis syndrome were used to select biomarkers in the level of metabolomics. Student's *t*-test was initially employed to detect metabolites that are of significant difference between model and sham operation group in swine as well as between UA patients and healthy control group. 21 metabolites in the plasma samples of swine model and 20 metabolites in patients with UA were found of significant value. Among which, 8 shared metabolites were found of low level expression in both swine model and UA patients. Then, independent student's *t*-test, principal component analysis (PCA), and hierarchical cluster analysis (HCA) were orderly applied to comprehend inner rules of variables in the data. The results indicated that the 8 shared metabolites can take place of the 21 or 20 metabolites in the classification of swine model with MI and patients with UA, which could be considered as a bridge connecting the mechanism basis and macrosyndromes of swine model with MI and UA patients. This research is for the first time trying to explore a bridge connecting the mechanism basis and macrosyndromes of human disease with experimental animal models by comparing the metabolites differences in human and animal plasma samples. But further researches need to be carried out, and more evidences need to be investigated to confirm this hypothesis.

## Supplementary Material

To evaluate the swine model with MI, echocardiography and hemorrheology were conducted. The results of echocardiography and hemorrheology of mini swine were given in Supplemental Table 1. In the clinical research, the demographic details of the 27 UA patients with blood stasis syndrome and 15 healthy controls were given in Supplemental Table 2. Different metabolites between model and sham operation in swine as well as between UA patients and healthy people were detected. Supplemental Table 3 listed the identified compounds and their retention time.

## Figures and Tables

**Table 1:** The shared metabolites in swine model and UA patients.

Number	Metabolite	*P* of swine model	Ascending (↑) or descending (↓)	*P* of UA patients	Ascending (↑) or descending (↓)
1	1,4-Benzenedica-rboxylic acid	0	↓	0.019387	↓
2	1,5-Anhydrogluc-itol	0.044	↓	0.005023	↓
3	2-Keto-d-gluconic acid	0.003	↓	0.056989	↓
4	Azelaic acid	0	↓	0.056989	↓
5	Heptanedioic acid	0	↓	0.031348	↓
6	Pentanedioic acid	0.019	↓	0.049076	↓
7	Ribitol	0.03	↓	0.010673	↓
8	Serine	0.001	↓	0.021365	↓

**Table 2:** The different metabolites in swine model and UA patients.

Animal model		Clinical patients	
Metabolite	*P*	Metabolite	*P*
2-Keto-1-gluconic acid	0.002	Tetracosanoic acid	0.017749
4-Pyrimidinamine	0.011	11-Eicosanoic	0.012502
Aminomalonic acid	0	4(1H) Pyridinone	0.003801
Butanedioic acid	0.008	Docosanoic acid	0.023955
Butanoic acid	0.032	Glucopyranoside	0.005449
Decanoic acid	0.015	Myo-Inositol	0.010473
D-Fructose	0	Octadecanoic acid	6.22*E*-05
Glutamine	0.002	citrazinic acid	0.003344
L-Cysteine	0.032	D-gluconic acid	0.015724
L-threonine	0.001	Glucose oxime	0.005449
L-Valine	0	Hexadecanoic acid	0.00186
Thiazolidine-4-carboxylic acid	0.044	Tetracosanoic acid	0.017749
Urea	0.001		
