# Supplementary material for: Metabolomics-Based Study of Clinical and Animal Plasma Samples in Coronary Heart Disease with Blood Stasis Syndrome
Source: Evid Based Complement Alternat Med. 2012 May 20;2012:638723. doi: 10.1155/2012/638723 (PMC3362859; doi:10.1155/2012/638723)
Supplement: Supplementary file 1 — To evaluate the swine model with MI, echocardiography and hemorrheology were conducted. The results of echocardiography and hemorrheology of mini swine were given in Supplemental Table 1. In the clinical research, the demographic details of the 27 UA patients with blood stasis syndrome and 15 healthy controls were given in Supplemental Table 2. Different metabolites between model and sham operation in swine as well as between UA patients and healthy people were detected. Supplemental Table 3 listed the identified compounds and their retention time. [file 638723.f1.doc]

**Supplemental Table 1:** **Echocardiography and hemorrheology of mini swine**

|  | Case(n=9) | Control(n=8) |
| --- | --- | --- |
| end-systolic volume | 17.41±8.60 | 10.02±2.66 |
| end-diastolic volume | 35.66±11.05 | 24.28±5.97 |
| end-systolic diameter | 2.78±0.41 | 2.07±0.08 |
| end-diastolic diameter | 3.93±0.41 | 3.28±0.26 |
| Anterior wall thickness of papillary muscle at end-systolic | 0.77±0.29 | 1.14±0.10 |
| Anterior wall thickness of papillary muscle at end-diastolic | 0.60±0.17 | 0.78±0.13 |
| Anterior wall thickness of apex at end-systolic | 0.55±0.28 | 1.12±0.16 |
| Anterior wall thickness of apex at end-diastolic | 0.49±0.21 | 0.77±0.13 |
| apex of the left ventricular anterior systolic wall thickening | 0.09±0.12 | 0.45±0.08 |
| septal thickness at the end-systolic | 0.78±0.17 | 0.98±0.08 |
| blood viscosity at a shear  rate of high | 0.62±0.55 | -0.31±0.80 |
| blood viscosity at a shear  rate of mid | 0.84±0.84 | -0.54±1.00 |
| blood viscosity at a shear  rate of low | 1.36±1.66 | -1.11±1.49 |

**Supplemental Table 2: Demographic detail of subjects**

|  | Case(n=27) | Control(n=15) P |
| --- | --- | --- |
| Age | 65±10 | 60±5 0.65 |
| Gender(Male/Female) | 14/13 | 8/7 0.93 |
| Smoking | 11(23%) | 4(27%) 0.51 |
| Hypertension | 16(59%) | 0(0%) _ |
| Hypercholesterolemia | 7(26%) | 0(0%) _ |
| Previous acute myocardial infarction | 2(7%) | 0(0%) 0.53 |
| Previous cerebral infarction | 6(22%) | 0(0%) _ |
| Use of beta blocker | 8(30%) | 0(0%) _ |
| Use of calcium channel antagonist | 8(30%) | 0(0%) _ |

**Supplemental Table 3: The list of identified compounds and their retention time**

| Order | Retention time | Metabolite name |
| --- | --- | --- |
| 1 | 6.419 | Propanoic acid |
| 2 | 7.33 | I-Alanine |
| 3 | 7.81 | Glycine |
| 4 | 7.87 | Butanoic acid |
| 5 | 8.25 | Ethoxyamine |
| 6 | 8.46 | 3,6-Dioxa |
| 7 | 8.729 | Butanoic acid |
| 8 | 9.12 | Cyclobutanone |
| 9 | 10.11 | L-Valine |
| 10 | 10.87 | Urea |
| 11 | 11.21 | Serine |
| 12 | 11.44 | Dipropylacetie acid |
| 13 | 11.64 | Phosphate |
| 14 | 12.13 | L-Isoleucine |
| 15 | 12.22 | L-Proline |
| 16 | 12.44 | Glycine |
| 17 | 12.73 | Butanedioic acid |
| 18 | 13.1 | Propanoic acid |
| 19 | 13.68 | 2-Butenedioic acid |
| 20 | 13.88 | L-Serine |
| 21 | 14.44 | 4-Pyrimidinamine |
| 22 | 14.51 | L-threonine |
| 23 | 15.08 | Pentanedioic acid |
| 24 | 16.37 | Decanoic acid |
| 25 | 16.61 | Aminomalonic acid |
| 26 | 16.75 | Thiazolidine-4-carboxylic acid |
| 27 | 17.11 | Butanedioic acid |
| 28 | 17.56 | Hexanedioic acid |
| 29 | 17.81 | L-Proline |
| 30 | 17.98 | L-Proline trans- |
| 31 | 18.4 | 2,3,4-Trihydroxybutyric acid |
| 32 | 18.67 | L-Cysteine |
| 33 | 18.82 | 2,3,4-Trihydroxybutyric acid |
| 34 | 18.94 | Benzoic acid |
| 35 | 19.04 | 2-Ethyl-propane |
| 36 | 19.29 | 2-Amino-n-caprylic acid |
| 37 | 19.88 | Heptanedioic acid |
| 38 | 20.07 | D-Ribofuranose |
| 39 | 20.27 | L-Phenylalanine |
| 40 | 22.01 | Octanedioic acid |
| 41 | 22.52 | Ribitol |
| 42 | 23.33 | 2-Keto-1-gluconic acid |
| 43 | 23.6 | Glutamine |
| 44 | 23.91 | 1,4-Benzenedicarboxylic acid |
| 45 | 24.13 | Azelaic acid |
| 46 | 24.21 | 2-Keto-d-gluconic acid |
| 47 | 24.39 | D-Fructose |
| 48 | 25.07 | L-Altrose |
| 49 | 25.19 | 1,5-anhydroglucitol |
| 50 | 25.6 | D-Fructose |
| 51 | 25.88 | D-Glucose |
| 52 | 26.02 | Glucopyranoside |
| 53 | 26.07 | Glucose oxime |
| 54 | 26.33 | Xylopyranose |
| 55 | 26.45 | Galactose oxime |
| 56 | 26.64 | L-Lysine |
| 57 | 26.72 | Glucitol |
| 58 | 26.95 | L-Tyrosine |
| 59 | 27.21 | Inositol |
| 60 | 27.79 | Glucopyranose |
| 61 | 28.08 | D-Gluconic acid |
| 62 | 28.72 | Gulose |
| 63 | 29.11 | Hexadecanoic acid |
| 64 | 29.77 | Myo-Inositol |
| 65 | 30.05 | Uric acid |
| 66 | 30.93 | Heptadecanoic acid |
| 67 | 32.18 | 11-cis-Octadecenoic acid |
| 68 | 32.65 | Octadecanoic acid |
| 69 | 33.84 | 4(1H)-Pyridinone |
| 70 | 35.55 | 11-Eicosenoic acid |
| 71 | 35.97 | Eicosanoic acid |
| 72 | 36.9 | citrazinic acid |
| 73 | 38.89 | D-Glucopyranoside |
| 74 | 39.03 | Docosanoic acid |
| 75 | 40.48 | Tricosanoic acdi |
| 76 | 40.85 | Maltose |
| 77 | 41.07 | Octadecanoic acid |
| 78 | 41.89 | Tetracosanoic acid |
| 79 | 45.77 | Cholesteral |
